# Supplementary material for: Alignment and quantification of ChIP-exo crosslinking patterns reveal the spatial organization of protein–DNA complexes
Source: Nucleic Acids Res. 2020 Aug 4;48(20):11215–26. doi: 10.1093/nar/gkaa618 (PMC7672471; doi:10.1093/nar/gkaa618)
Supplement: gkaa618_Supplemental_File [file gkaa618_supplemental_file.docx]

Supplement to “Alignment and quantification of ChIP-exo crosslinking patterns reveal the spatial organization of protein-DNA complexes”

Naomi Yamada^1^, Matthew J. Rossi^1^, Nina Farrell^1^, B. Franklin Pugh^1^, Shaun Mahony^1^*****

^1^ Center for Eukaryotic Gene Regulation, Department of Biochemistry & Molecular Biology, The Pennsylvania State University, University Park, PA 16802.

[mahony@psu.edu](mailto:mahony@psu.edu)


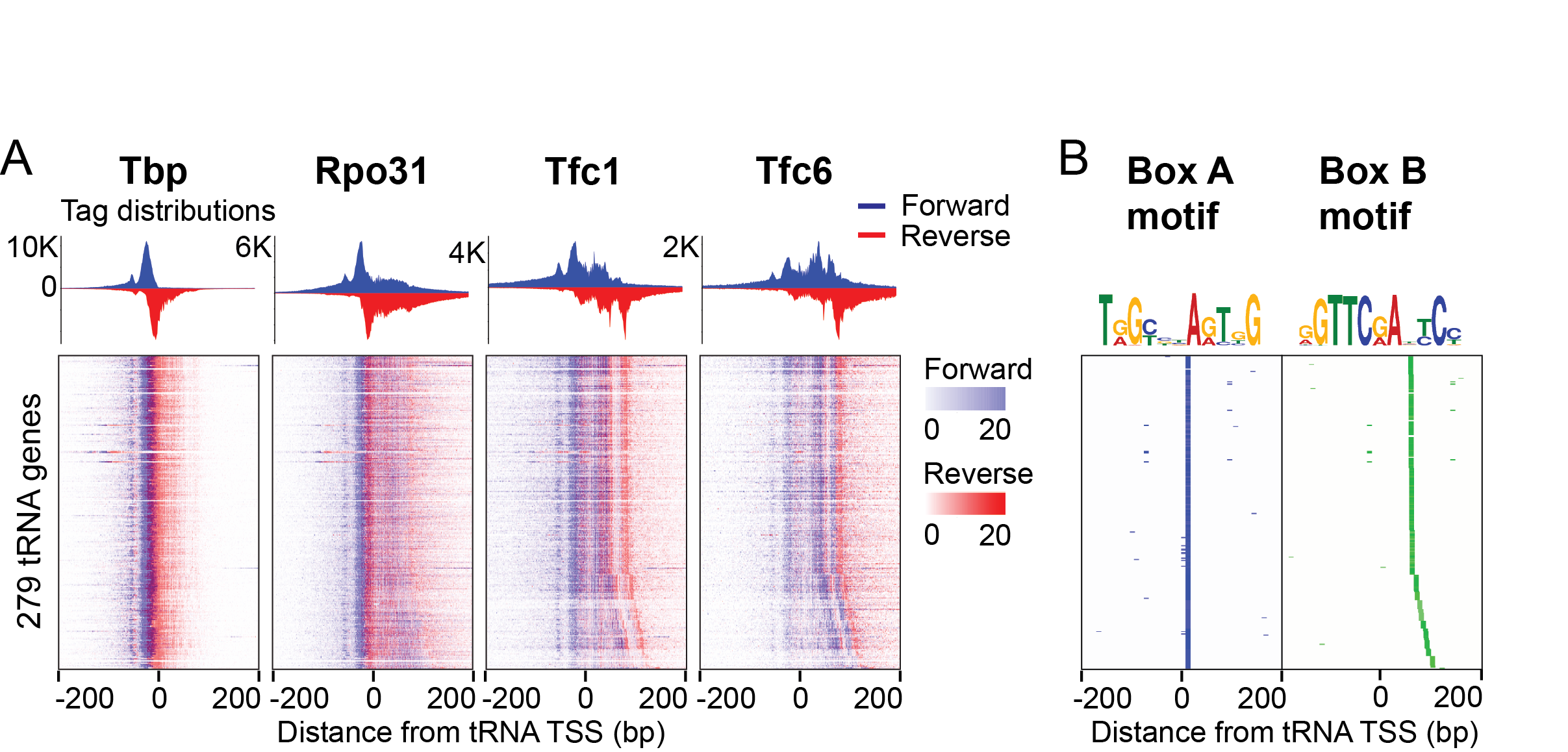


**Figure S1.** Related to Figure 3C, E. **A)** ChIP-exo heatmaps centered around tRNA TSS are shown for TBP (TFIIIB), Rpo31 (Pol III), Tfc1 (TFIIIC τA), and Tfc6 (TFIIIC τB). The heatmaps are sorted by increasing tRNA gene length. **B)** Positions of Box A and Box B motifs centered around tRNA TSSs.


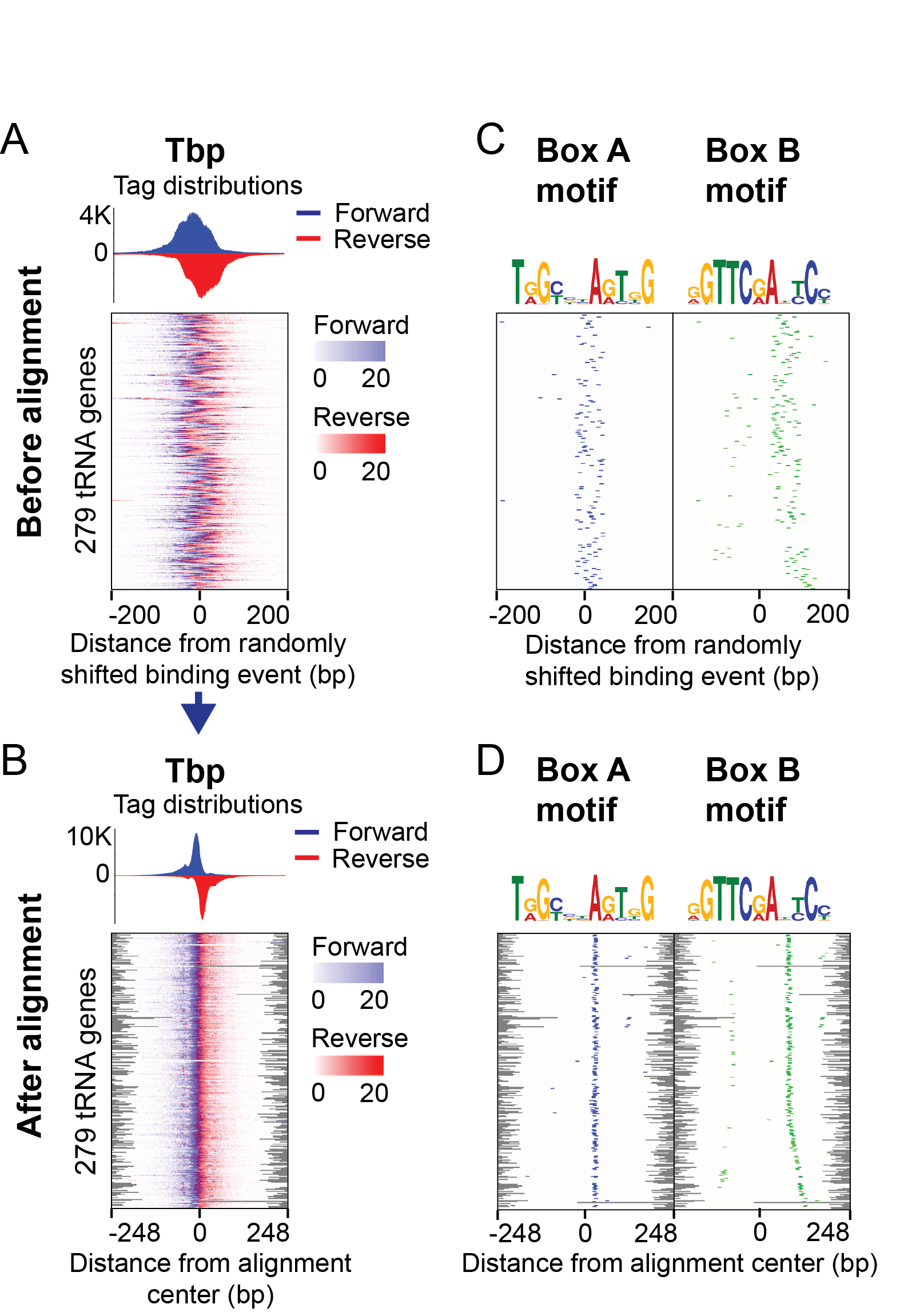


**Figure S2.** Related to Figure 3. **A), B)** Tag distribution of TBP (TFIIIB) ChIP-exo data around randomly shifted tRNA genes before (A) and after (B) the alignment of a single TBP ChIP-exo data. Alignment start positions are randomly shifted +- 30 bp around tRNA TSSs. The heatmaps are sorted by increasing tRNA gene length. **C), D)** Relative positions of Box A and Box B motifs before (C) and after (D) the alignment.


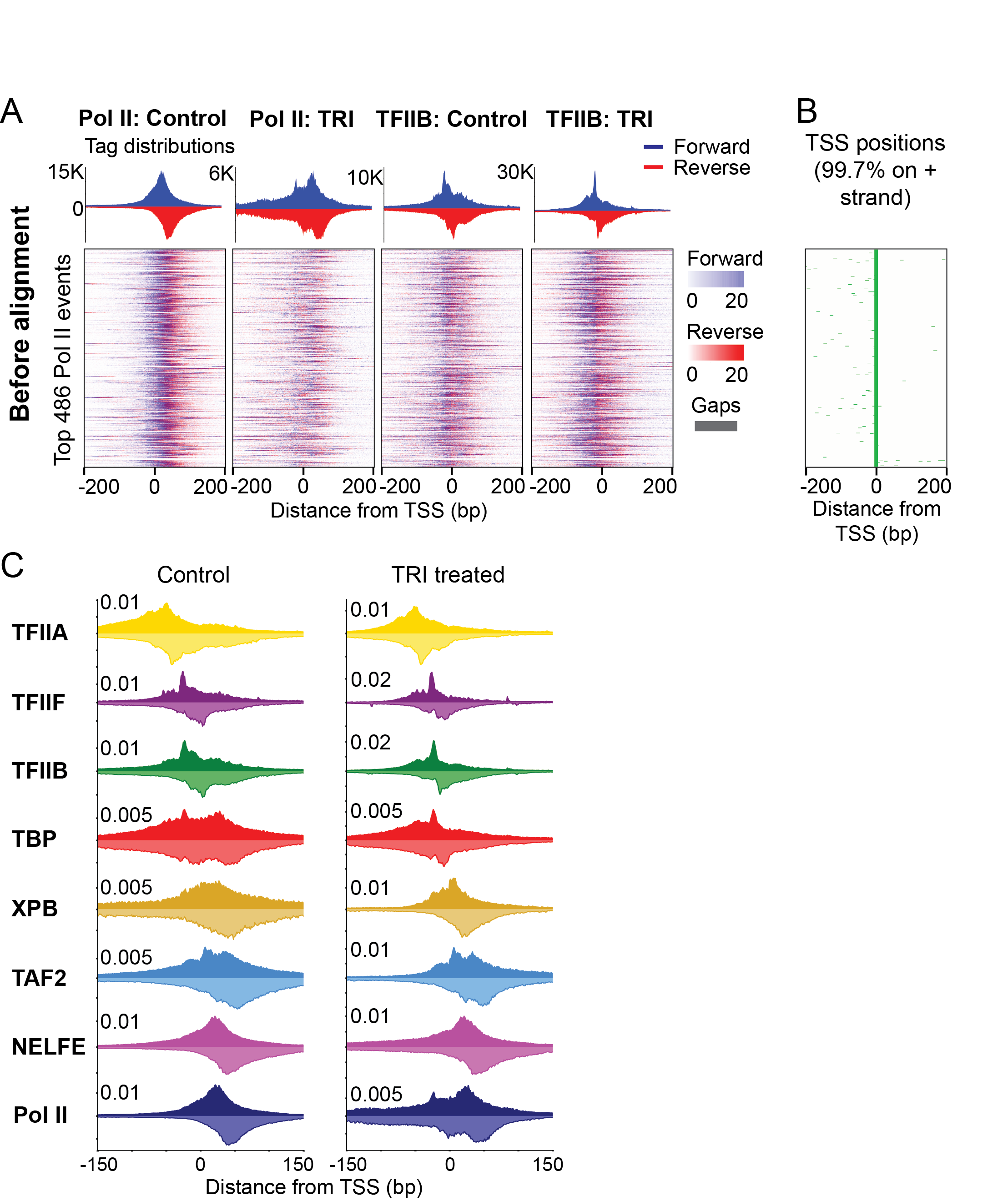


**Figure S3.** Related to Figure 5A, 5B, 5C. **A**) ChIP-nexus profiles for Pol II and TFIIB in control and TRI treatment conditions centered around TSSs closest to the top 486 Pol II binding events. **B**) Annotated *Drosophila* gene TSS positions (refGene). **C**) ChIP-nexus tag patterns of eight factors with or without TRI treatment.


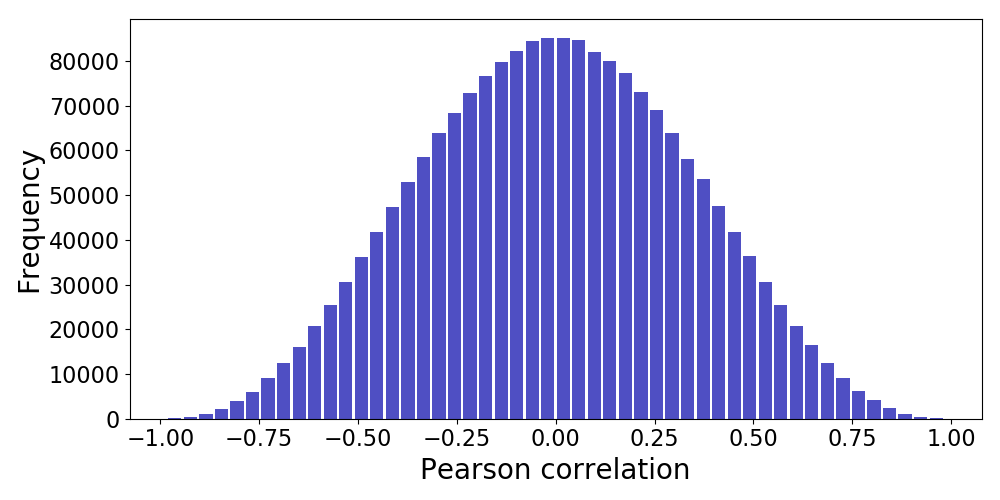


**Figure S4.** Distribution of per-base Pearson correlation for independent variables. We generated random values ranging between 0 to 1 representing normalized read counts in an array size of 1,400 for positive and negative strands across five experiments. Then, Pearson correlation was calculated according the formula in the main manuscript using these simulated datasets.


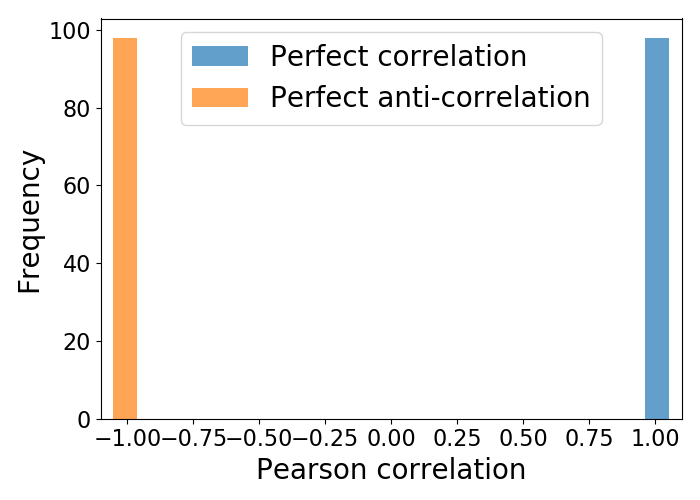


**Figure S5.** Perfect correlation and anti-correlation calculated using Pearson correlation similarity score. Random numbers between 0 to 1 are generated in a 100 bp window for positive and negative strands across five conditions. Perfect correlation (score=1) was generated using two arrays of same values. Perfect anti-correlation (score=-1) was generated using an array with complementary values (1- value) ranging between 0 to 1.


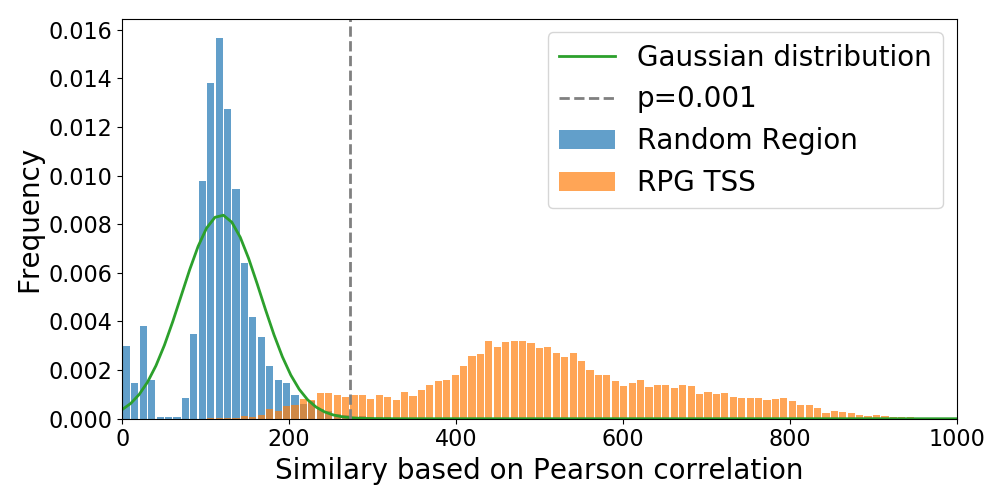


**Figure S6.** Distribution of per-region similarities for random regions (blue bar) and RPG TSS (orange bar). Gaussian distribution is fit to similarity scores from random regions (green line). P value of 0.001 is shown using grey dotted line.


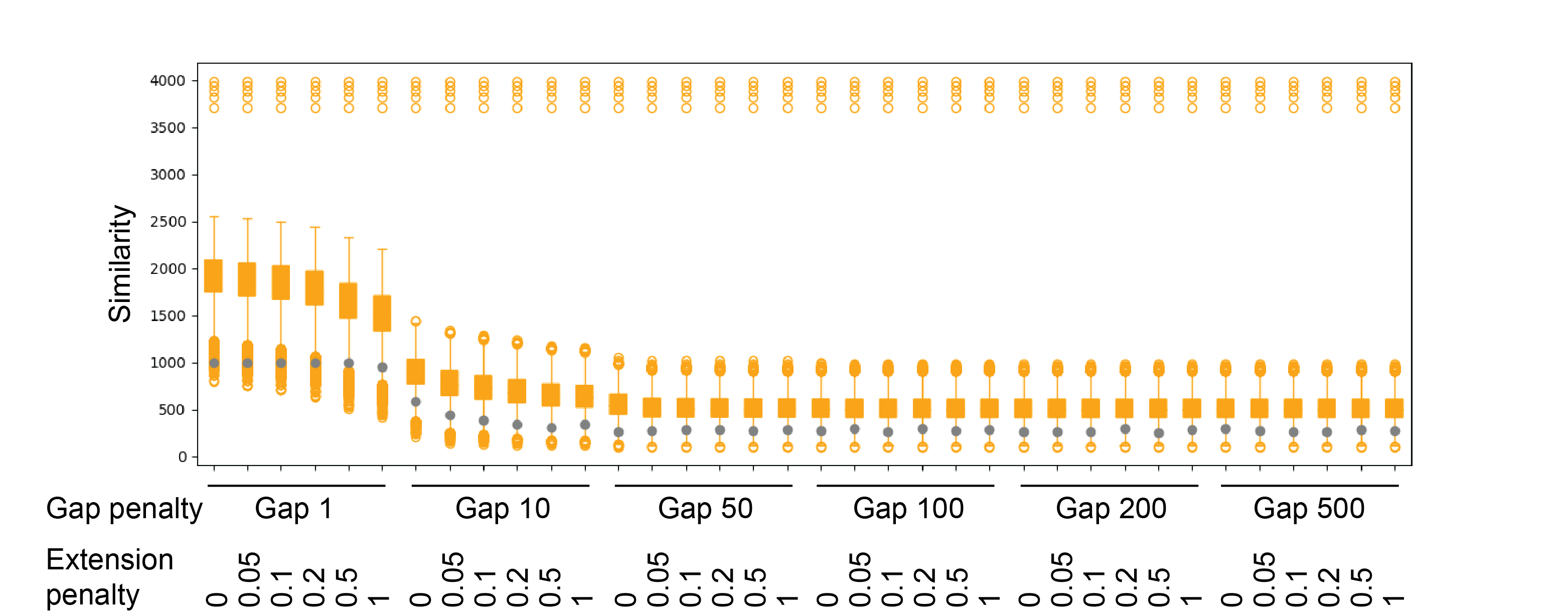


**Figure S7.** Similarity scores over different gap penalty and gap extension penalties. Scores using RPG TSS as starting positions are shown in orange color box plots. Gaussian distribution is fit to scores from random regions. P-value of 0.001 using random regions are shown as grey dots.
